# Indian psychiatry: Research and international perspectives

**DOI:** 10.4103/0019-5545.69205

**Published:** 2010-01

**Authors:** Roy Abraham Kallivayalil, Rakesh K. Chadda, Juan E. Mezzich

**Affiliations:** Department of Psychiatry, Co-operative Medical College, Cochin, India; 1 All India Institute of Medical Sciences, New Delhi, India; 2 International Center for Mental Health, Mount Sinai School of Medicine, New York University, New York, USA

**Keywords:** Indian, psychiatry, research, mental health

## Abstract

Indian psychiatry has many contributions to the world psychiatry to its credit. These include active participation in the international scientific organizations, research, and also creation of the manpower resources in many other countries. India has been an active partner in the research initiatives of the World Health Organization and the World Psychiatric Association. Research by the Indian psychiatrists played an important role in recognition of the entity of acute and transient psychotic disorders, some culture bound syndromes like Dhat syndrome and understanding the role of families in care of schizophrenia and course and outcome of schizophrenia.

Indian Psychiatry has had a considerable influence on World Psychiatry, especially by promoting a broad conceptualization of health, which is enshrined in the World Health Organization’s (WHO’s) constitution. India has also contributed to the development of the cultural bases of psychiatry through the work of many eminent researchers, such as Venkoba Rao, Kapur, Wig, Varma, Shekhar Saxena, Dinesh Bhugra and others. Contributions of many clinical scientists of Indian origin are also noteworthy in the development of biological psychiatry. Indian psychiatrists have also contributed immensely to the research, academics, and organizational activities of the World Psychiatric Association (WPA) and the World Association for Social Psychiatry (WASP). World Psychiatry has in turn influenced Indian Psychiatry by providing a global framework within which Indian psychiatrists and institutions are making specific contributions, and also by facilitating the development of collaborative links within and across the North–South and East–West divides.

Modern psychiatry in India developed as an extension of British psychiatry, as most of the psychiatrists who started modern psychiatry in India were trained in the United Kingdom. The training of psychiatry in various medical schools and the mental health institutes also had strong influences of British and American psychiatry. However, most of the current generation of psychiatrists have been primarily trained in India, although some might have received further training abroad. The Indian Council of Medical Research (ICMR) as well as the WHO have made an important contributions funding many quality research projects in the country. India has made tremendous contributions to world psychiatry. The contributions are in various areas including creating manpower resources around the world, especially in the English speaking countries, in research, therapeutics, international psychiatric associations, and the WHO. The contributions of the country can be traced back to some ancient Indian medical texts, which at that time had given elaborate classification of psychiatric disorders, as well as teachings of religious texts, which have relevance in psychotherapy.

This chapter discusses the contributions of Indian psychiatrists to world psychiatry starting from the ancient period and also briefly on how World Psychiatry has influenced Indian Psychiatry. It is not possible to summarize all the contributions in a single article. The authors have attempted to highlight some important areas. It is possible that some important contributions may have been missed inadvertently.

## CONTRIBUTIONS OF ANCIENT INDIA

Mental health being an important component of health was recognized even by the ancient physicians of India including Charaka and Sushruta, more than two thousand years ago. Ayurveda, is the ancient Indian text on medicine. The concept of mind and a description of psychosis, ‘*unmad*’, have been given in the *Vedas* sometime between 5000 and 10000 BC.[1] A discourse by Lord Krishna to the historical warrior ‘Arjuna’ in the battle field of Mahabharata, available in text form, in the Gita, is an excellent treatise on psychotherapy. Gita is available in a translated version, in English. Many Indian psychiatrists like Vidya Sagar and A Venkoba Rao have emphasized on the use of teachings from the Gita in psychotherapy.[2] Professor Vidya Sagar often used anecdotes from the Gita in his group sessions with patients and their families. Buddha’s teachings to his disciples also have tremendous psychotherapeutic potential.[3]

Ayurveda has given an elaborate classification of mental diseases as neuroses or *chittodwaga* (anxiety disorders), *unmada* (psychoses), *apasmara* (convulsive disorders, epilepsy) and *attattvabhinivesa* (obsessive disorders); while *mada* (intoxication), *murcha* (syncope), and *sanyasa* (coma) are considered to be psychosomatic diseases. Unmada or psychosis, is described by Charaka as ‘the perversion of the mind, intellect, conscience, behavior, and conduct.’ According to Charaka, there are five types of *unmada: vata, pitta, kapha, samnipatika* (combined vitiation of all the three *doshas*) and exogenous. *Samnipatika* is considered incurable. Exogenous insanity is different from the insanity caused by vitiated *doshas*, and is believed to be caused by karma and the ‘…effects of sinful activities in past life’ Ayurveda also gives a personality classification based on interaction between ‘*doshas*’ and ‘*Gunas*’.[4] The origin of the yogic method of relaxation can also be traced to the ancient India. Recently, Suchitra *et al*.,[5] have given a tridimensional model of psychosis (unmada) based on Ayurveda.

## CONTRIBUTIONS OF THE INDIAN PSYCHIATRISTS IN THE LAST 50 YEARS

In the last 50 years, Indian psychiatrists have conducted research in many areas including schizophrenia and related psychotic disorders, mood disorders, anxiety disorders, substance use disorders, epidemiology, community psychiatry, child and adolescent psychiatry, disaster-related mental health problems, and many other areas. Psychiatric units in the general hospitals are one of the main providers of health care in India. They provide scope for close integration of psychiatry with other specialties, and have also helped in reducing the stigma associated with mental disorders.

### Schizophrenia and related disorders

India was a member country in the WHO’s multicentric International Pilot Study of Schizophrenia (IPSS; World Health Organization, 1979) and the Determinants of the Outcome of Severe Mental Disorders (DOSMED) study.[6] Both provided convincing evidence for a better outcome in India (along with other less industrialized countries) than in the West. This finding of a good outcome of treatment also emerged in the Chandigarh studies.[7,8] The discovery of a good prognosis of schizophrenia, in Indian patients with schizophrenia, was further confirmed by the ICMR’s multisite study of the Factors Affecting the Course and Outcome of Schizophrenia (SOFACOS), which had an extended follow-up to 10 years.[9,10] Indian psychiatrists have also done extensive work on acute psychoses. Short-term follow-up also showed the delineation of acute psychosis as distinct from schizophrenia and affective disorders,[11] and was responsible for the acceptance of the concept of acute and transient psychotic disorders.

Another important difference in which India differs from the West is that more than 90% of the patients of schizophrenia stay with their families. Families face substantial burden due the care-giving role. Indian psychiatrists have worked on the area of expressed emotions, family burden, family interventions, and development of support groups. One study has shown lesser expressed emotions in the Indian families.[12] The recognition of the importance of families has led to the creation of facilities for families to stay with patients in some centers, such as the National Institute of Mental Health and Neurosciences in Bangalore, and the Christian Medical College in Vellore. This facilitates the extensive participation of families in therapeutic programs.[13] Coping by families has also received some attention, with particular reference to religious coping methods.[14,15] There have also been studies exploring the needs of patients of schizophrenia.[16] Qualitative research in the area of care-giving is also being undertaken.[17,18]

Psychiatrists in India have also been working on contemporary issues in schizophrenia. There have been many important contributions by the Indian Psychiatrists in areas such as the metabolic syndrome in schizophrenia. One recent study reported the low prevalence of the metabolic syndrome and obesity in untreated patients of schizophrenia.[19] Others have worked on the role of the second generation antipsychotics in causing the metabolic syndrome.[20] There has also been work exploring the genetics of schizophrenia as well as the biological abnormalities. Venkatsubramanian *et al*.,[21] have recently been able to identify deficits in the corpus callosum in antipsychotic naïve patients of schizophrenia.

### Culture bound syndromes

One seminal article by Professor NN Wig has introduced the concept of the Dhat syndrome more than 50 years ago,[22] which can be considered as a form of somatization. It is a culture-bound syndrome, characterized by a belief of passing semen in urine, which leads to loss of sexual vigor and is accompanied by a number of vague somatic symptoms as well as secondary anxiety and depressive symptoms.[23] The patients present with a characteristic profile of illness behavior with hypochondriasis and denial.[24] The syndrome has been mainly reported from South East Asia, although some authors have contested its occurrence only in this population and its being a culture-bound syndrome.[25]

Excessive vaginal discharge is another common complaint reported by women from the Indian subcontinent and has attracted the interest of the Indian psychiatrists. The complaint is often accompanied by vague somatic symptoms, which indicates the underlying psychological stress. In the local parlance, the complaint is referred to as leucorrhea, ‘safed pani’, ‘shwet prader’ and many other local names are used.[26,27] In one community survey, nearly 15% of the women complained of excessive vaginal discharge.[28] Koro has also been reported both as isolated cases as well as an epidemic from India.[29,30]

### Mood disorders

Mood disorders have attracted the interest of the Indian psychiatrists since the beginning. The initial study was on the symptomatology of depression, especially the finding of less prevalence of guilt in Indian patients as compared to the west.[31–33] Indian patients suffering from depression have also been reported to show higher scores on items such as poor appetite, hypochondriasis, diurnal variation, and psychomotor retardation; and lower scores on anxiety and middle insomnia.[33] Some workers have carried out studies on the role of melatonin in depression.[34]

Social issues such as disability associated with depression, untreated depression, and attitudes toward depression still remain an area of interest to the Indian psychiatrists. Poverty and poor physical health have been recognized as important risk factors in old age depression, whereas, good social support is protective.[35] Chakraborty *et al*.,[36] identified erroneous beliefs about antidepressants in patients with depression, which can interfere with compliance, thus highlighting the need of psycho-education.

A number of Indian psychiatrists have worked in the area of neurocognition, burden and care-giving, and quality of life in bipolar disorder. A number of Indian researchers have tried to identify sources of burden and care-giving models in bipolar disorder.[15,37,38] Trivedi *et al*.,[39] have identified impairments in executive functioning in the euthymic state, in patients with bipolar disorder. The same group of authors also found poor executive function of vigilance in the normal siblings of patients with bipolar disorder.[40] Impairment in executive functioning and soft signs have also been detected in euthymic states, in bipolar patients, in some Indian studies.[41,42] Chand *et al*.,[43] could not find any differences in the quality of life of patients with bipolar disorder, while in a euthymic state, as compared to the normal controls.

In recent years, research has also been carried out to study the brain functioning in bipolar disorder. Bhardwaj *et al*,[44] by using a (99 m) Tc-ECD SPECT, found reduced perfusion in the left frontal area, and left anterior cingulated and parietal cortices in patients with mania, and lowered rCBF in the anterior temporal regions bilaterally, as also in the left parietal area, in patients with bipolar depression.

### Common mental disorders

There has been an extensive study on various common mental disorders including anxiety disorders, somatoform disorders, and depression in primary care. A number of researchers in India have studied psychiatric diagnoses in somatizing patients. Depressive, anxiety, and somatoform disorders are the common diagnoses, but a substantial number of patients are placed under unspecified categories of these disorders.[45,46] Chaturvedi and Bhandari[47] have studied illness behavior patterns in somatizing patients. Younger patients were found to have disease phobia and preoccupation with the disease. Srinivasan and Suresh[48] used a method of screening for nonspecific symptoms, to identify psychiatric morbidity in primary care. Symptoms such as weakness of body or mind, headache, fatigability, and burning sensations are commonly reported by Indian patients.[46,49] In another study on somatization and depression, Raguram *et al*.,[50] concluded that the tendency to perceive and report distress in psychological or somatic terms is influenced by various social and cultural factors, including the degree of stigma associated with particular symptoms. Patel *et al*,[51] have studied the somatic and psychological models of common mental disorders. Duddu *et al*,[52] have hypothesized alexithymia in the background of somatization and depression in Indian patients.

### Person-centered medicine

Indian Psychiatry has been more recently collaborating and advancing the concept of person-centered psychiatry. We have been associated with the Institutional Program for Person-Centered Psychiatry of the World Psychiatric Association initiated by Juan E Mezzich (President of World Psychiatric Association 2005 – 2008). The concept, ‘Toward person-centered medicine: from disease to patient to person,’ has been discussed by Mezzich *et al*.[53] Roy Abraham Kallivayalil and Trivedi have been roped in as the steering committee members of this Program and are participating in person-centered diagnostic developments. Preparing educational curricula for person-centered clinical care, its integration with General Health Care, training Primary Care Physicians in person-centered medicine, and integration with the National Mental Health Program are agendas for the future.

### Indian psychiatry and world psychiatric association

India is a member of the WPA and has been closely involved with its activities. Advancing the regional development of world psychiatry through the studies of many Indian colleagues is notable. Trivedi was the Zonal representative in 2005 – 2008 and Mohandas in the current period. They have been involved with coordinating WPA activities in the South Asia Region. Roy Abraham Kallivayalil is the co-chairman of the WPA Preventive Psychiatry Section, and a book on ‘Suicide Prevention — a handbook for community gate-keepers’ was published in 2009,[54] by the National Alliance for Mental Health, India, as an important activity of the Section.

### India and social psychiatry

Promoting the development of social psychiatry through the contributions of many scholars and through the Indian Association of Social Psychiatry (IASP) has been remarkable. Varma, Sridhar Sharma, Rakesh K Chadda, Roy Abraham Kallivayalil, and others have led the Social Psychiatry movement in India. IASP has been the participant in the recent WPA Survey on ‘Reducing the treatment gap for mental disorders.’[55]

### India and the mental health legislation

India has made significant strides in providing humane treatment for the mentally ill. The Mental Health Act (1987) annulled many archaic provisions of the Indian Lunacy Act (1912). However, further improvements in the mental health legislation have become necessary and imperative and these have been discussed in a recent article by Kallivayalil and Trivedi.[56]

### Indian psychiatrists in other parts of the world

India has contributed to the world psychiatry in another way by being a supplier of an extensive workforce of psychiatrists and other mental health professionals to the developed world. A recent report[57] estimates that 4687 psychiatrists of Indian origin are currently registered in countries like UK, USA, Australia, New Zealand, and other countries. The number is much more than the Indian psychiatrists working in India. They have also made a significant impact in these countries, in areas of service as well as research. Many of them have reached important academic positions in the Universities and have also made important contributions to the international agencies like the WHO. The assumption of office of Shekhar Saxena as the Director, Department of Mental Health and Substance Abuse at WHO, Geneva, is worth special mention. One of the psychiatrists of Indian origin — Dinesh Bhugra — was recently elected President of the Royal College of Psychiatrists in the United Kingdom.

## CONCLUSION

Indian Psychiatry is on the path of rapid development. Vast improvements in mental health delivery have been made during the last few decades, but the paucity of resources, lack of adequate mental health personnel, and the general apathy toward mental health are obstacles that need to be overcome. Despite many severe constraints, India has made the most significant contribution to the world, among South Asian countries. The liaison with world psychiatry has also progressed further by affording links to global medical organizations such as the World Psychiatric Association, World Association for Social Psychiatry, World Medical Association, World Federation for Mental Health, and others, which represent new opportunities and horizons for development and fulfillment.

## References

[1] Gautam S (1999). Mental health in ancient India and its relevance to modern psychiatry: Presidential address. Indian J Psychiatry.

[2] Venkoba Rao A (1980). Gita and mental sciences. Indian J Psychiatry.

[3] Vidya S (1973). Challenge of our times: Presidential Address. Indian J Psychiatry.

[4] Dube KC (1979). Nosology and therapy of mental illness in Ayurveda. Comp Med East West.

[5] Suchitra SP, Devika HS, Gangadhar BN, Nagarathna R, Nagendra HR, Kulkarni R (2010). Measuring the tridosha symptoms of unmāda (psychosis): A preliminary study. J Altern Complement Med.

[6] Sartorius N, Jablensky A, Korten A, Ernberg G, Anker M, Cooper JE (1986). Early manifestations and first-contact incidence of schizophrenia in different cultures. Psychol Med.

[7] Kulhara P, Wig NN (1978). The chronicity of schizophrenia in North West India: Results of a follow-up study. Br J Psychiatry.

[8] Kulhara P (1994). Outcome of schizophrenia: some transcultural observations with particular reference to developing countries. Eur Arch Psychiatry Clin Neurosci.

[9] Thara R, Rajkumar S (1992). Gender differences in schizophrenia: Results of a follow-up study from India. Schizophr Res.

[10] Thara R, Srinivasan TN (1997). Outcome of marriage in schizophrenia. Soc Psychiatry Psychiatr Epidemiol.

[11] Susser E, Varma VK, Malhotra S, Conover S, Amador XF (1995). Delineation of acute and transient psychotic disorders in a developing country setting. Br J Psychiatry.

[12] Leff J, Wig NN, Gosh A, Bedi H, Menon DK, Kuipers L (1987). Influence of relatives expressed emotion on the course of schizophrenia in Chandigarh. Br J Psychiatry.

[13] Thara R, Padmavati R, Srinivasan TN (2004). Focus on psychiatry in India. Br J Psychiatry.

[14] Rammohan A, Rao K, Subbakrishna DK (2002). Religious coping and psychological wellbeing in carers of relatives with schizophrenia. Acta Psychiatr Scand.

[15] Chadda RK, Singh TB, Ganguly KK (2007). Caregiver Burden and Coping: A prospective study of relationship between burden and coping in caregivers of patients with schizophrenia and bipolar affective disorder. Soc Psychiatry Psychiatr Epidemiol.

[16] Kulhara P, Avasthi A, Grover S, Sharan P, Sharma P, Malhotra S (2009). Needs of Indian schizophrenia patients: An exploratory study from India. Soc Psychiatry Psychiatr Epidemiol.

[17] Aggarwal M, Avasthi A, Kumar S, Grover S (2009). Experience of caregiving in schizophrenia: A study from India. Int J Soc Psychiatry.

[18] Ganguly KK, Chadda RK, Singh TB (2010). Caregiver burden and coping in schizophrenia and bipolar disorder: a qualitative study. Am J Psychiatr Rehabil.

[19] Padmavati R, McCreadie RG, Tirupati S (2010). Low prevalence of obesity and metabolic syndrome in never-treated chronic schizophrenia. Schizophr Res.

[20] Saddichha S, Manjunatha N, Ameen S, Akhtar S (2008). Metabolic syndrome in first episode schizophrenia - a randomized double-blind controlled, short-term prospective study. Schizophr Res.

[21] Venkatasubramanian G, Jayakumar PN, Reddy VV, Reddy US, Gangadhar BN, Keshavan MS (2010). Corpus callosum deficits in antipsychotic-naïve schizophrenia: Evidence for neurodevelopmental pathogenesis. Psychiatry Res.

[22] Wig NN, DeSouza A, DeSouza DA (1984). Psychiatric research in India. Psychiatry in India.

[23] Chadda RK, Ahuja N (1990). Dhat syndrome: A culture bound neurosis of the Indian subcontinent. Br J Psychiatry.

[24] Chadda RK (1995). Dhat syndrome - Is it a distinct clinical entity? A study of illness behaviour characteristics. Acta Psychiatr Scand.

[25] Sumathipala A, Siribaddana SH, Bhugra D (2004). Culture-bound syndromes: The story of dhat syndrome. Br J Psychiatry.

[26] Chaturvedi SK (1993). Abnormal illness behaviour and somatization due to leucorrhoea. Psychopathology.

[27] Patel V, Andrew G, Pelto PJ (2008). The psychological and social contexts of complaints of abnormal vaginal discharge: A study of illness narratives in India. J Psychosom Res.

[28] Patel V, Pednekar S, Weiss H, Rodrigues M, Barros P, Nayak B (2005). Why do women complain of vaginal discharge? A population survey of infectious and pyschosocial risk factors in a South Asian community. Int J Epidemiol.

[29] Duuta D, Phookun HR, Das PD (1982). The Koro Epidemic In Lower Assam. Indian J Psychiatry.

[30] Chowdhury AN (1992). Koro Social Response (URBAN): A Longitudinal Study Of North Bengal Koro Epidemic. Indian J Psychiatry.

[31] Bagadia VN, Jeste DV, Dave KP, Doshi SU, Shah LP (1973). Depression: A clinical study of 233 cases. Indian J Psychiatry.

[32] Sethi BB, Prakash R, Arora U (1980). Guilt and hostility in depression. Indian J Psychiatry.

[33] Ananth J, Engelsman F, Ghadirian AM, Wohl M, Shamasundara P, Narayanan HS (1993). Depression and guilt in Indian and North American patients: A comparative study. Indian J Psychiatry.

[34] Venkoba Rao A, Srinivasan V, Parvati Devi S (1983). Urinary melatonin in depression. Indian J Psychiatry.

[35] Rajkumar AP, Thangadurai P, Senthilkumar P, Gayathri K, Prince M, Jacob KS (2009). Nature, prevalence and factors associated with depression among the elderly in a rural south Indian community. Int Psychogeriatr.

[36] Chakraborty K, Avasthi A, Kumar S, Grover S (2009). Attitudes and beliefs of patients of first episode depression towards antidepressants and their adherence to treatment. Soc Psychiatry Psychiatr Epidemiol.

[37] Chakrabarti S, Gill S (2002). Coping and its correlates among caregivers of patients with bipolar disorder: A preliminary study. Bipolar Disord.

[38] Nehra R, Chakrabarti S, Kulhara P, Sharma R (2005). Caregiver-coping in bipolar disorder and schizophrenia--a re-examination. Soc Psychiatry Psychiatr Epidemiol.

[39] Trivedi JK, Dhyani M, Sharma S, Sinha PK, Singh AP, Tandon R (2008). Cognitive functions in euthymic state of bipolar disorder: An Indian study. Cogn Neuropsychiatry.

[40] Trivedi JK, Goel D, Dhyani M, Sharma S, Singh AP, Sinha PK (2008). Neurocognition in first-degree healthy relatives (siblings) of bipolar affective disorder patients. Psychiatry Clin Neurosci.

[41] Goswami U, Sharma A, Khastigir U, Ferrier IN, Young AH, Gallagher P (2006). Neuropsychological dysfunction, soft neurological signs and social disability in euthymic patients with bipolar disorder. Br J Psychiatry.

[42] Kolur US, Reddy YC, John JP, Kandavel T, Jain S (2006). Sustained attention and executive functions in euthymic young people with bipolar disorder. Br J Psychiatry.

[43] Chand PK, Mattoo SK, Sharan P (2004). Quality of life and its correlates in patients with bipolar disorder stabilized on lithium prophylaxis. Psychiatry Clin Neurosci.

[44] Bhardwaj R, Chakrabarti S, Mittal BR, Sharan P (2010). A single photon emission computerized tomography (SPECT) study of regional cerebral blood flow in bipolar disorder. World J Biol Psychiatry.

[45] Saxena S, Nepal MK, Mohan D (1988). DSM-III axis I diagnoses of Indian psychiatric patients with somatic symptoms. Am J Psychiatry.

[46] Chadda RK, Bhatia MS, Shome S (1991). Physical symptoms in psychiatry - diagnostic uncertainties and clinical characteristics. Indian J Psychiatry.

[47] Chaturvedi SK, Bhandari S (1989). Somatization and illness behaviour. J Psychosom Res.

[48] Srinivasan TN, Suresh TR (1991). The nonspecific symptom screening method: Detection of nonpsychotic morbidity based on nonspecific symptoms. Gen Hosp Psychiatry.

[49] Chaturvedi SK, Michael A (1993). Do social and demographic factors influence the nature and localisation of somatic complaints?. Psychopathology.

[50] Raguram R, Weiss MG, Channabasavanna SM, Devins GM (1996). Stigma, depression, and somatization in South India. Am J Psychiatry.

[51] Patel V, Pereira J, Mann AH (1998). Somatic and psychological models of common mental disorder in primary care in India. Psychol Med.

[52] Duddu V, Isaac MK, Chaturvedi SK (2003). Alexithymia in somatoform and depressive disorders. J Psychosom Res.

[53] Mezzich J, Snaedal J, van Weel C, Heath I (2010). Toward person-centered medicine: From disease to patient to person. Mt Sinai J Med.

[54] Kallivayalil RA, Punnoose VP (2009). Suicide Prevention- a handbook for community gate –keepers.

[55] Patel V, Maj M, Flisher AJ, De Silva MJ, Koschorke M, Prince M (2010). World Psychiatry. WPA.

[56] Kallivayalil RA, Trivedi JK, Tripathi A (2009). Social factors and forensic psychiatry in India. Curr Opin Psychiatry.

[57] Jenkins R, Kydd R, Mullen P, Thomson K, Sculley J, Kuper S (2010). International migration of doctors, and its impact on availability of psychiatrists in low and middle income countries. PLoS One.

